# Saliva Based Liquid Biopsies in Head and Neck Cancer: How Far Are We From the Clinic?

**DOI:** 10.3389/fonc.2022.828434

**Published:** 2022-03-21

**Authors:** Aditi Patel, Shanaya Patel, Parina Patel, Vivek Tanavde

**Affiliations:** ^1^ Biological and Life Sciences, School of Arts and Sciences, Ahmedabad University, Ahmedabad, India; ^2^ Bioinformatics Institute, Agency for Science Technology and Research (ASTAR), Singapore, Singapore

**Keywords:** head and neck cancer, liquid biopsy, saliva, biomarker, circulating tumor nucleic acids, extracellular vesicles, metabolomics

## Abstract

Head and neck cancer (HNC) remains to be a major cause of mortality worldwide because of confounding factors such as late-stage tumor diagnosis, loco-regional aggressiveness and distant metastasis. The current standardized diagnostic regime for HNC is tissue biopsy which fails to determine the thorough tumor dynamics. Therefore, due to the ease of collection, recent studies have focused on the utility of saliva based liquid biopsy approach for serial sampling, early diagnosis, prognosis, longitudinal monitoring of disease progression and treatment response in HNC patients. Saliva collection is convenient, non-invasive, and pain-free and offers repetitive sampling along with real time monitoring of the disease. Moreover, the detection, isolation and analysis of tumor-derived components such as Circulating Tumor Nucleic Acids (CTNAs), Extracellular Vesicles (EVs), Circulating Tumor Cells (CTCs) and metabolites from saliva can be used for genomic and proteomic examination of HNC patients. Although, these circulatory biomarkers have a wide range of applications in clinical settings, no validated data has yet been established for their usage in clinical practice for HNC. Improvements in isolation and detection technologies and next-generation sequencing analysis have resolved many technological hurdles, allowing a wide range of saliva based liquid biopsy application in clinical backgrounds. Thus, in this review, we discussed the rationality of saliva as plausible biofluid and clinical sample for diagnosis, prognosis and therapeutics of HNC. We have described the molecular components of saliva that could mirror the disease status, recent outcomes of salivaomics associated with HNC and current technologies which have the potential to improve the clinical value of saliva in HNC.

## Introduction

Head and Neck Cancer (HNC) is the sixth most prevalent cancer worldwide attributed to etiological factors like tobacco and alcohol consumption, HPV infections and to a certain extent genetic predisposition (1–3). Despite advancements in diagnostic and therapeutic regime, the overall survival of HNC patients has remained dismal for over four decades. Conventional diagnostic strategies comprise of physical examination, imaging techniques such as computed tomography (CT) scan, Ultrasound (US), magnetic resonance imaging (MRI) and tissue biopsies followed by histopathological analysis. Till date, tissue biopsy is the most commonly used method for diagnosis; however, this technique is invasive, quite challenging, painful, time-consuming, and potentially risky for the patient. Moreover, the intra-tumoral and metastatic heterogeneity remains undetected, affecting the specificity, sensitivity and accuracy of assessment (4). Therefore the ‘liquid biopsy approach’ that focuses on detecting tumor-derived components in circulatory fluids for the diagnosis, screening and prognosis of cancer (5) is becoming increasingly important. Liquid biopsies are anticipated to demonstrate high accuracy in terms of representation of tumor genome landscape and mutations. They also provide reproducibility and feasibility of real-time therapeutic monitoring while being minimally invasive and cost effective (6). For HNC cancers, serum, plasma and saliva have been identified as the most frequently used sources for liquid biopsies (7).

**Figure 1:**
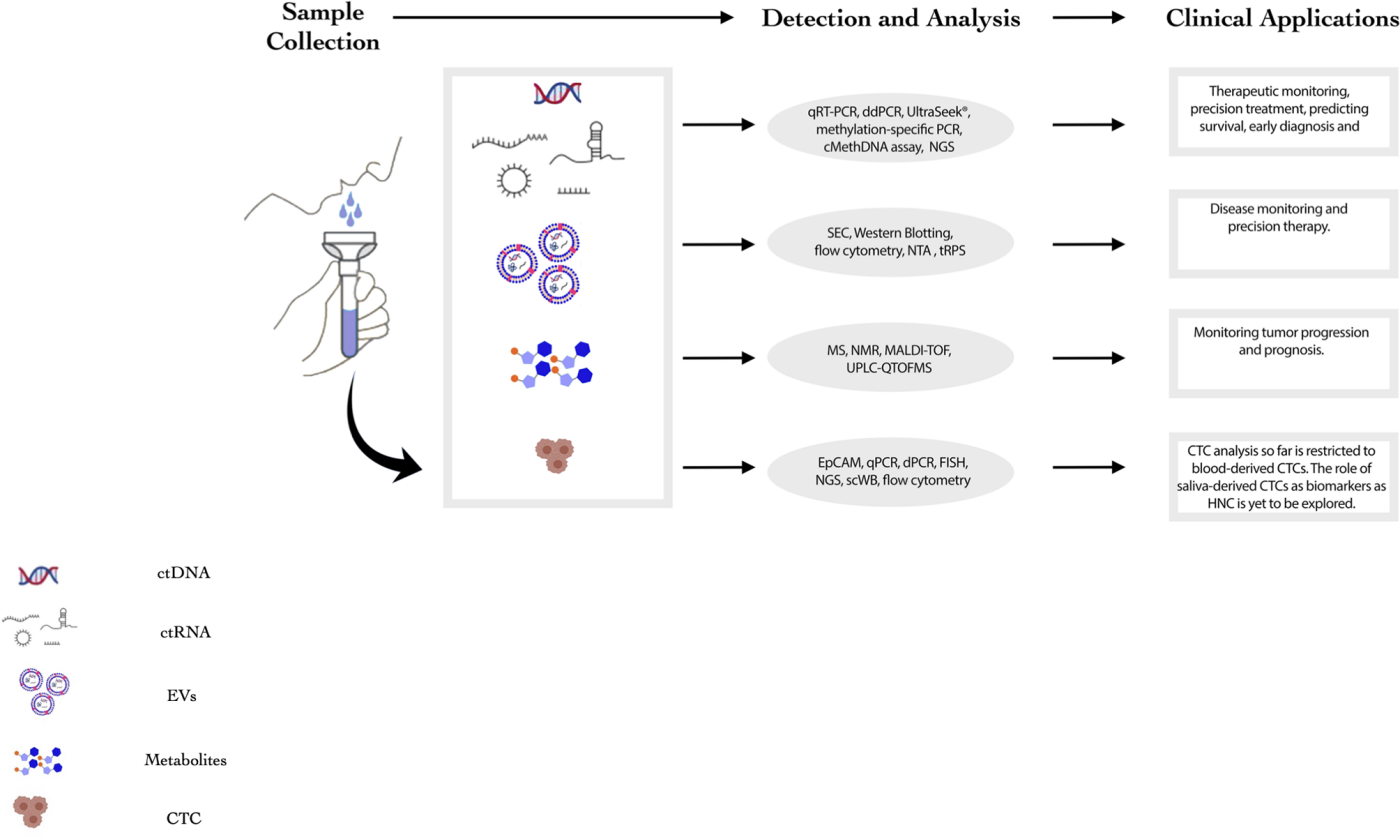
Summary of salivary components that can potentially act as biomarkers for HNC. This figure summarizes the current landscape of salivary components that may act as biomarkers for HNC. The detection and analysis techniques and clinical applications for each component are mentioned in the figure. **(**NMR: Nuclear Magnetic Resonance, MS: Mass Spectrometry, HNC: Head and Neck Cancer, ddPCR: Droplet Digital Polymerase Chain Reaction, qPCR: quantitative Polymerase Chain Reaction, NGS: Next Generation Sequencing, SEC: Size-Exclusion Chromatography, NTA: Nanoparticle Tracking Analysis).

Saliva as a potential source for liquid biopsy of HNC patients has several advantages compared to other body fluids as it (i) reflects any genomic, epigenomic, proteomic and physiological/pathological alterations in the oral cavity, larynx and pharynx; (ii) serves as a non-invasive, inexpensive, easier and more accessible screening tool (8); and (iii) provides the opportunity for real-time monitoring of HNC patients by having the flexibility of repetitive sampling and larger volumes for examination without the requirement of trained medical staff for collections (8–10). Despite the potential value in utilizing saliva derived biomarkers as diagnostic tool, its clinical utility is limited due to some challenges. Primarily, the complex composition of saliva comprises of various non-tumorigenic components hampering the ability to detect biomolecules of tumor origin. Moreover, relative contribution of different subsites into the salivary milieu makes the identification of HNC specific markers difficult (11). However, the potential utility of saliva as a liquid biopsy tool for diagnosis, prognosis and therapeutic monitoring of HNC is being extensively explored. Presently, the most common components for liquid biopsy of HNCs comprise cell-free tumor nucleic acids (DNA, mRNA and miRNAs), extracellular vesicles, circulating tumor cells (CTCs) and salivary metabolites (**Figure 1**). This review encompasses the recent developments, technologies, clinical applications and limitations of saliva derived biomarkers in HNC diagnosis, prognosis, and therapeutics.

## Circulatory Tumor Nucleic Acids

Circulatory tumor nucleic acids (ctNAs) are fragments of cell-free genomic/viral DNA and/or RNA that are shed by tumor cells through passive processes like necrosis and apoptosis or *via* active mechanisms like spontaneous release of nucleic acids in systemic circulation (12, 13). These fragments of circulating tumor DNA (ctDNA) and RNA (ctRNA), are found in various body fluids including saliva. They reflect the genetic information of the bulk tumor and reflect clonal heterogeneity and tumor evolution. The rate at which these circulatory nucleic acids release into circulation depends on the tumor’s location, vascularity, scale, resulting in variability across patients (14). Analysis of ctDNA relies more on identifying and targeting certain tumor specific mutations and understanding the epigenetic landscape, whereas ctRNA emphasizes on identifying novel or differential expression patterns of messenger RNA (mRNAs), microRNA (miRNAs), and long ncRNAs (lncRNAs) as a potential salivary biomarker. ctRNA based biomarkers probably gives better dynamic insights about cell-state and regulation as compared to ctDNA biomarkers.

Detection and analysis of ctNAs is quite challenging. Currently, real-time PCR (qRT-PCR), digital droplet PCR (ddPCR) and UltraSeek^®^ (Agena Bioscience) mass-spectrometry-based PCR method are the most widely used techniques, as it helps in optimizing samples with low ctNA concentration in HNCs. ddPCR is still the most preferred method demonstrating higher sensitivity, specificity and multiplexing capacity (15, 16). Further, techniques such as methylation-specific PCR (17, 18), methylation on beads (19), and cMethDNA assay (20, 21) are used to detect the difference in methylation patterns on promoter of ctDNA in HNC patient samples. PCR based techniques are preferred when there are low number of target regions (≤ 20 targets), limited sample input and when there is limited assessment of tumor heterogeneity or identification of known variants. Next Generation Sequencing (NGS) methods such as CAPP-Seq (cancer personalized profiling by deep sequencing), TAm-Seq (tagged amplicon deep sequencing), Safe-Seq (safe sequencing system), and AmpliSeq are being used to isolate and capture ctNAs; each with relatively higher strengths in sensitivity, specificity and scalability (22–24). These NGS techniques can detect both known and unknown tumor-specific mutations and analyze differential expression patterns of single markers or a panel of markers. Targeted NGS methods are less time-consuming, result in fewer wastage of resources and offer a higher discovery rate, thus aiding in identification of novel variants. Despite the current limitations, these techniques have demonstrated potential to detect and isolate smaller concentrations of ctDNA from saliva, thus opening new avenues for clinical applications (25). With technological advancements, higher specificity and sensitivity of ctDNA detection could effectively increase their clinical applications. Nonetheless development of cost-effective NGS assays is crucial for their widespread clinical utility (26, 27).

## Circulatory Tumor DNA (ctDNA)

ctDNA represents a trivial fraction (<1%) of whole cfDNA shed from tumor cells into the circulation. However, this small subpopulation is believed to reflect the somatic mutations and genomic landscape from primary tumors that can be useful in early diagnosis and risk prediction of HNC. Recently, few studies have emphasized utilizing ctDNA derived from saliva in early detection of cancer. Wang et al., conducted a comprehensive analysis of somatic mutations (*TP53, CDKN2A, NRAS*, *NOTCH1, PIK3CA, FBXW7*, and *HRAS*) and HPV (HPV16 and 18) genes in saliva and plasma of 93 HNC patient samples comprising of oral cavity, oropharynx, larynx, and hypopharynx subsites. The study demonstrated detection of ctDNA at 96% rate irrespective of the tumor size, stage and location. Moreover, recurrence post-surgery was observed in majority of patients having these somatic mutations. In OSCC patients, the detection rate of ctDNA was higher in saliva as compared to plasma, indicating that salivary ctDNA can be used for OSCC detection (28).. Similarly, p53 mutation in exon 4 codon 63 was detected in saliva of early stage OSCC patients (93.33% of cases, p<0.05) with a similar detection rate as patient tumor samples (29). However, Perdomo et al. reported that, targeted mutation detection approach failed to demonstrate significant concordance in detecting *TP53* mutations from tumor and saliva derived ctDNA. El Naggar *et al.* and Spafford et al., detected microsatellite instability and loss of heterozygosity at certain chromosomes in oral mucosal cells from HNC patients and saliva (p < 0.001) with different sensitivity and specificity based on sample size and sampling subsites (30, 31). Moreover, genetic alterations in *PMAIP1* and *PTPN1* genes had the potential to discern HNC patients from healthy individuals (32). Collectively, these studies suggest that assessment of somatic mutations from salivary ctDNA can be an effective non-invasive substitute to tissue biopsy for early diagnosis, disease surveillance and prognosis of HNC patients. However, multiple mutation detection-based studies with standardized protocols and larger cohort of patients will be required for clinical translation (33) of this approach. Low yields of ctDNA after purification from saliva is also a key limiting factor. To increase the efficacy and sensitivity of salivary ctDNA as a biomarker, specific ctDNA panels need to be designed that can help detect and monitor HNC cases in real-time and a cost-effective manner.

Several studies have highlighted the importance and feasibility of detecting epigenetic alterations in ctDNA from body fluids and its immense diagnostic potential. Promoter hyper-methylation of genes such as *EDNRB* (κ = 0.60), *KIF1A (κ = 0.64), NID2*(κ = 0.60), and *HOXA9 (κ = 0.60)* in salivary DNA have shown potential utility for early detection of oral cancer patients (34–36). Few studies have demonstrated a significant clinical correlation between hypermethylation in promoter region of salivary ctDNA with prognosis and risk prediction in HNC patients. Specifically, methylated gene loci were identified in both tissue and preoperative saliva samples and could serve as a classifier to differentiate between preoperative and postoperative samples for HNC patients (37). Analogous to this, Carvalho et al. indicated that detection of promoter hypermethylation of either or all genes (*TIMP-3, CCNA1, DCC, MGMT, MINT-31, DAPK p16*) in pre-treated salivary DNA could effectively predict poor survival (HR=2.8; 95% CI=1.2–6.5; p=0.016) and recurrence (HR=12.2; 95% CI=1.8–80.6; p= 0.010) of HNC patients (38).These findings suggest that elevated ctDNA hypermethylation patterns have the potential to predict disease aggressiveness, overall survival rate and therapeutic monitoring and surveillance of HNC patients (**Table 1**). As we thrive towards the development of epigenetic-based diagnostic tests, we need to consider the challenges that come along with it. One of the major challenges: Given the epigenetic plasticity in non-cancerous cells, we need to develop tools that can filter the false positive signals and enhance the specificity and sensitivity of these assays making them more translatable.

**Table 1:** ctDNA biomarkers for HNSCC.

Marker	Type of Marker	Findings	Sample Size	Reference
E7 (HPV16 and HPV18), TP53, PIK3CA, CDKN2A, FBXW7, HRAS, and NRAS	Diagnostic	The sensitivity of detection of ctDNA increased when both saliva and plasma assays were combined (96% of the samples). Moreover, oral cavity tumor ctDNA was preferentially enriched in saliva as opposed to ctDNA from other sites.	93	(28)
CDKN1A and DDB2	Post-treatment monitoring	Salivary CDKN1A and DDB2 were significantly upregulated post-treatment in HNSCC patients and the rate of upregulation was correlated with the received treatment dose.	8	(39)
HPV DNA	Prognostic	Salivary HPV DNA levels in patients with LR HPV^+^ OPSCC were correlated to total tumor burden. A rise of salivary HPV DNA was correlated with recurrence and a fall in HPV DNA levels was observed during treatment. Higher levels of plasma HPV cfDNA were associated with poor prognosis.	21	(40)

Collectively, somatic mutations and methylation patterns of salivary DNA could be utilized as potential biomarkers and prognosticators in HNC. This approach can accelerate the diagnosis and risk prediction of HNC and pave the path for improved patient outcomes by monitoring their therapeutic response.

## Circulatory Tumor RNA (ctRNA)

Analysis of transcriptomic profiles of circulating body fluids is a widely explored method for early cancer detection and several studies have shown significant association of the transcriptome with disease progression. Several studies have demonstrated the association of salivary mRNAs with development and detection of HNC. Li et al. demonstrated a significant 3.5-fold elevation in OSCC saliva with significant sensitivity (91%) and specificity (91%) (P < 0.01) of transcripts of salivary *SAT, IL8, S100P, IL1B, OAZ1, DUSP1* and *HA3*, in oral cancer patients as compared to their healthy counterparts (41). David Elashoff and colleagues (42) substantiated the effectiveness of these biomarkers in a larger patient cohort (382 patients), suggesting the potential role of salivary mRNA markers in oral cancer detection. With respect to individual marker performance across the five cohorts, the increase in *IL8* and *SAT* was statistically significant(p<0.02). The validation of these biomarkers in larger patient cohorts shows their feasibility in the discrimination of OSCCs from healthy controls (42). Transcript level expression of tumor suppressor gene transgelin was observed to be significantly elevated in saliva of OSCC patients as compared to the normal counterparts. The salivary gene expression levels were in concordance with the tumor tissue and associated with overall survival (p=0.011) of patients, demonstrating its immense potential as a promising biomarker and an independent prognosticator in OSCC (43). *HPV-16* has also been identified as a major etiological factor responsible for HNSCC tumorigenesis. *HPV-16* mRNA showed a significantly altered expression in salivary rinses of HNSCC patients with a simultaneous effect on p16(INK4a), a known tumor suppressor having a vital role in regulating the cell cycle(p<0.05) (44). Thus, the expression pattern of different salivary mRNAs correlates with various important clinical parameters including tumor progression, differentiation, and overall survival. More importantly, the expression of salivary mRNA depicts an independent prognosis factor for HNC, suggesting that salivary mRNA might be a potential biomarker for early detection of HNC and predicting the prognosis for HNC patients.

Tumor derived circulating RNA profile is complex as it comprises of distinctive components such as noncoding RNAs (e.g., lncRNA and piwi-interacting RNAs) and microRNAs (miRNAs). Alterations in miRNA and lncRNAs expression can be exploited to investigate their potential in differentiating HNC patients from healthy volunteers (45, 46), given the fact that substantial research has been conducted in exploring the diagnostic and prognostic potential of ncRNAs derived from saliva of HNC patients (**Table 2**) (55). Various saliva-derived circulatory miRNAs such *as miR-139-5p* in TSCC (49) *miR-3612, miR-650, miR-4259, miR-937-5p* and *miR-4478* in NPC (51) and *miR-125a, miR-200a*, and *miR-21* have been identified as plausible biomarkers for different subsites of HNC (46, 48). In a preliminary study, expression of 314 salivary miRNAs was assessed in OSCC patients in comparison to their healthy counterparts. *miR-200a* and *miR-125* were observed to be significantly down regulated (p<0.05) in the patient cohort as compared to the healthy volunteers. This study emphasized that salivary miRNAs were stable in saliva and could be utilized in early detection of oral cancers (46). These findings were validated by Wiklund and colleagues demonstrating that differential expression of *miR-200a* and *miR-375* along with promoter methylation of miR-200c-141 in oral rinses and saliva of OSCC patients can be utilized for early detection of oral cancers (56). The potential role of circulatory miRNAs in effectively monitoring tumor progression, therapeutic response and recurrence have been reported in several studies – (i) Salivary miR-21 is associated with T-stage classification (p=0.02) (54) (ii) and miR-136 expression showed significant correlation with complete remission cases (AUC=0.904 CI=0.75-1 P<0.05) (25) Moreover, a preliminary study conducted by Greither et al. demonstrated differential expression of salivary miR-200a (p=0.036) and miR-93 (p=0.047) in HNSCC patients post-radiotherapy (50). Similarly, another study identified significant correlation between increased expression of salivary miR-15a-5p and disease-free survival in post-intensity modulated radiotherapy patients (HR=0.25; 95% CI=0.05-0.78; p<0.016) (57). These studies highlighted the utility and efficacy of saliva-based miRNA biomarkers in predicting therapeutic response despite the significant alterations in salivary components post-radiation.

**Table 2:** Circulating miRNA markers for HNSCC.

Marker	Type of Marker	Findings	Tumor Sample Size	Author
miR-31	Diagnostic	Upregulation of salivary miR-31 in OC patients.	35	(47)
miR-21 and miR-184	Diagnostic	Highly significant upregulation of miR-21 and miR-184 (*P* < 0.001) in OSCC and PMD samples as compared to healthy controls.	40	(48)
miR-139-5p	Diagnostic	Significant downregulation of salivary miR-139-5p in TSCC patients as compared to healthy controls. Levels returned to normal after treatment (surgery).	25	(49)
miR-93 and miR-200a	Treatment monitoring	Increase in expression of miR-93 and miR-200a in OSCC patients 12 months after radiotherapy thereby highlighting their potential as biomarkers for post-radiation treatment monitoring in HNSCC patients.	33	(50)
miR-937-5p, miR-650, miR-3612, miR-4478, miR-4259, miR-3714, miR-4730, miR-1203, miR-30b-3p, miR-1321, miR-1202 and miR-575	Diagnostic	Identified 12 miRNAs that were significantly downregulated in the saliva of NPSCC patients and could potentially serve as diagnostic biomarkers.	22	(51)
miR-let-7a-5p and miR- 3928	Diagnostic	Salivary miR- let-7a-5p and miR- 3928 were significantly downregulated in HNSCC patients as compared to healthy controls. Both of these miRNAs showed significant specificity and sensitivity in differentiating between healthy controls and HNSCC patients.	12	(52)
miR-24-3p	Diagnostic	Significantly high expression of exosomal miR-24-3p was observed in saliva of OSCC patients.	30	(53)
miR-21 and miR-31	Diagnostic	Upregulation of salivary miR-31 and miR-21 in patients with severe dysplasia relative to healthy controls. Leucoplakia had the most significant upregulation of the aforementioned markers out of all the lesions.	36	(54)

The other arm of ncRNAs are long non-coding RNAs (lncRNAs) which are approximately more than 200 nucleotides long and are not translated into protein. Considering their inevitable role in tumor progression and metastasis, signatures of saliva derived lncRNAs have been explored as probable biomarkers for monitoring disease progression of OSCC. A pilot study has reported measurable levels of HOTAIR and MALAT lncRNAs in the saliva of OSCC patients (58). Furthermore, these elevated levels were associated with nodal metastasis ascertaining its potential as a predictive marker.

Recently, circular RNAs (circRNAs) have attracted attention globally, because of their stability (owing to the circular structure) in comparison to lncRNAs and miRNAs (45). Various circRNAs secreted into the saliva of HNC patients regulate several biological and physical processes (59). A study found differential expression of 32 salivary circRNAs in OSCC patients as compared to matched controls. The upregulation of hsa-circ-0001874 clinically correlated with tumor grade and staging. Expression level of hsa-circ-0001971 was associated with TNM stage. Further, these circRNAs could also differentiate OSCC from oral leucoplakias (AUC of 0.895) (60). These findings prompt towards their potential role as diagnostic biomarkers for OSCC; however, additional investigation on circRNAs as probable non-invasive biomarkers for HNCs will be needed to assess their prognostic and diagnostic value.

The use of salivary ctRNAs as biomarkers for detection, disease surveillance, therapy response, and prognosis sound promising but a major limitation of salivary RNA quantification is the risk of RNA degradation due to the presence of enzymes including RNases in the saliva. This in turn affects the quality of RNA extracted thereby increasing the false-positive and false-negative detection rates. Moreover, the risk of sample contamination with blood from the oral mucosa and lesions due to inflammation are other limiting factors. Multicentric preclinical/clinical studies with standardized protocols are required to verify the existing findings before establishing the clinical utility of circulatory RNAs.

## Extracellular Vesicles

Extracellular vesicles (EVs) are 30-200 nm membrane encapsulated organelles that are secreted by cells into the extracellular space in response to various physiological conditions such as proteases, growth factors, apoptotic signals, biomechanical shear and stress conditions (61, 62). Developing evidence suggests that tumor-derived EVs enable the tumor bulk to manipulate its microenvironment as they have the potential to mediate intercellular communication by transporting their molecular cargo (DNA, RNA and protein) to local or distant sites through circulatory fluids (63).

Conventional EV isolation techniques are dependent on their physical and biological properties such as size, density and surface marker expression (64). Conventionally utilized techniques for EV isolation and purification based on size include filtration and size-exclusion chromatography (SEC) whereas immune-affinity capture method identifies the EV population based on surface markers. Currently, the widely used methods for EV isolation are ultracentrifugation and/or differential centrifugation and polymer precipitation method which is commonly used in commercially available kits. Recently developed microfluidics-based technologies for EV isolation comprise of antibody-functionalized microfluidic channels (65), nanoscale size-based filtration (66) and spiral inertial microfluidic devices (67). After isolation, western blotting and flow cytometry using surface protein markers CD9, CD63, CD81, Alix, TSG101, are the most conventionally used analytical methodologies for characterisation of EVs (68–71). Nanoparticle Tracking Analysis (NTA) that works on the principle of determining Brownian motion of the particles is another extensively used technique and has higher resolution as compared to flow cytometry (72). Similar to NTA, Tunable Resistive Pulse Sensing (tRPS) is an emerging technology that estimates the EV concentration based on the particle movement and flow rates in fluid cells corresponding to the pulses/voltage applied (73, 74). However, the clinical applicability of tRPS remains to be challenging considering the heterogenous size of the EV population. Several techniques have been explored for isolation and characterisation of EVs using various patient samples; however, sensitivity and specificity of these techniques in terms of clinical utility for liquid biopsies requires comprehensive standardization of protocols and larger patient cohort studies.

Various findings have revealed that saliva harbors ample numbers of EVs, the components of which differ based on the physiological or pathological state of an individual (75). Some of the advantages of salivary EVs as compared to serum and plasma derived EVs are – (i) the collection process is non-invasive; (ii) they contain less protein content that makes their identification and quantification simpler (76, 77); and (iii) they do not undergo coagulation which stimulates a persistent secretion of EVs from platelets, thus altering the composition of circulating EVs (78). Recently, the possibility of potential biomarkers from circulatory EVs derived from saliva of HNC patients is gaining interest (**Table 3**). On comparing plasma and salivary EVs derived from oral cancer patients it was found that salivary EVs were concomitantly elevated as the plasma derived EVs and demonstrated a clinical association with tumor staging (p<0.01) and loco-regional aggressiveness (p<0.01) (81). These results are in corroboration with previous studies showing that salivary EVs from oral cancer patients have an irregular morphology, are greater in size and formed more aggregates as compared to EVs from normal controls (82–84).

**Table 3:** Exosomal biomarkers for HNSCC.

Author	Type of Marker	Findings	Tumor Sample Size	Reference
***RNA***				
miR-21	Diagnostic	Hypoxic OSCC derived exosomes expressed higher levels of miR-21 and the expression was closely associated with lymph node metastasis and T-stage of the cancer.	108	(79)
miR-302b-3p, miR-517b-3p, miR-512-3p and miR-412-3p	Diagnostic	miR-302b-3p and miR-517b-3p were exclusively expressed in salivary EVs isolated from OSCC samples. miR-412-3p and miR-512-3p were significantly upregulated in salivary EVs of OSCC patients as compared to healthy controls (*p* < 0.02).	21	(9)
miR-24-3p	Diagnostic	Salivary exosomal miR-24-3p levels significantly increased in OSCC patients. miR-24-3p interacts with *PER1* thereby promoting the proliferation of OSCC.	49	(53)
***Proteins***				
MMP-9, myosin-9 (NMMHC II-a), complement C3, S100A9, complement factor B (CFB), Rab GDI and complement C4-B	Diagnostic	Differentially expressed proteins were reported in salivary OSCC samples as compared to control samples. Out of the group of 38 proteins that were identified only in OSCC samples, 5 were identified in patients without any lesions.	21	(80)

Recent studies have found a significant role of salivary EV derived non-coding miRNAs as potential biomarkers for early diagnosis, prognosis and therapeutic targets in HNC patients given their stability within the EV and ability to regulate both oncogenes and tumor suppressor genes. Significantly, elevated levels of *miR-21, miR-494-3p, miR-412-3p, miR-184, miR-27a-3p*, and *miR-512-3p* (p<0.05) were observed in salivary exosomes derived from OSCC patients compared to the control cohort (9, 85). A recent study demonstrated that salivary miR-24-3p was enriched in OSCC and tongue cancer patients and could significantly increase the proliferation of these cells (53). Collectively these findings suggest that salivary exosomal miRNAs can be an asset for convenient and non-invasive sampling as well as pave way for early diagnosis, disease monitoring and therapeutic response evaluation in various HNC subsites (86–88).

Recent studies have reported that EVs contain long non coding RNAs (lncRNAs), however their expression has not been explored extensively in salivary EVs. High expression of a subset of lncRNAs, including HOTAIR, has been reported in the saliva of metastatic HNC patients. Thus, besides miRNAs, lncRNAs in salivary EVs could be a valued prognostic and diagnostic asset for HNC (89, 90).

The discovery of tumor associated proteins in saliva is accredited to high-throughput mass spectrometry screening of patient samples. From these studies, a series of protein biomarkers has been detected in salivary EVs for OSCC, such as *LGALS3BP, PKM1/M2, A2M, MUC5B, IGHA1, HPa, and PIP* (80, 91). Moreover, these tumor-associated proteins have been reported to be involved in multiple signaling pathways, including metal transport, cell proliferation, and tumor immune responses (80). Additionally, exosomal *EGFR, ANXA1* and programmed cell death (PD)-1/PD-ligand 1 (*PD-L1*) pathway (tumor suppressor in HNSCC) have been identified as potential biomarkers for predicting prognosis and therapeutic monitoring in tumor derived exosomes of HNSCC patients (92).

There are several benefits of EVs as compared to ctDNA and CTCs. However, a wide range of isolation and analysis techniques for EVs and lack of universally accepted EV reference standards are some of the major hurdles for developing diagnostic assays to enumerate EVs from patient samples. Moreover, interference from hemolytic, lipaemic and platelet contaminated samples and issues with sample stability compromise the reproducibility of EV detection, modify EV’s physical and biological properties and affect their purity and recovery rate (93). Hence, developing a consistent external quality assessment (EQA) scheme involving application of strict but attainable sample requirements for assays, establishing standardized collection and storage environments that can minimize EV degradation and applying standard methods of EV characterization and enumeration is needed.

Salivary EVs have enormous potential for future diagnostic and therapeutic modalities, but this potential needs to be underpinned with solid scientific groundwork. A comprehensive understanding about the mechanism of how cancer cells utilize EVs to promote carcinogenesis may direct the advancement of novel therapies for HNC.

## Salivary Metabolomics

Metabolomics focusses on identification and quantification of small metabolites produced during the process of metabolism from biological samples including body fluids, cells, and tissues. Increasing evidence has highlighted the importance and potential clinical utility of metabolomics in differentiating between HNC patients and controls using bio-fluids such as saliva, plasma and serum of HNC patients.

Currently, mass spectrometry (MS) and nuclear magnetic resonance (NMR) are the most frequently used procedures for screening salivary metabolites for early diagnosis and therapeutic monitoring of HNC patients (94). For salivary metabolite-based analysis, solution state NMR is the most preferred technique and protons (1H) are the most commonly analyzed NMR-active nuclei (95). One of the major advantages of this technique is that simple steps such as centrifugation are sufficient and no other pre-processing is required for sample preparation (96). The utility of MS techniques such as matrix-assisted laser desorption ionization (MALDI) in combination with time-of-flight (TOF) is being explored in salivary metabolomics as it can provide a high-throughput profile from a small sample volume without the requirement of a separation step (97, 98). Apart from this, liquid chromatography MS (LC-MS) is a frequently used technique for screening saliva samples for metabolites. Capillary electrophoresis MS (CE-MS) is an emerging technique that utilizes high voltages to induce an electrophoretic flow of ions through a capillary (20–200 µm i.d.) using very small sample volumes (10-100 nanolitre). The unique advantage of CE-MS is its ability to boost the range of detectable polar metabolites; however complex assembly and the high possibility of capillary blockage are confounders (99, 100). Therefore, it is crucial to develop a standard protocol for processing saliva samples for metabolomic analysis for successful clinical translation.

Identification of salivary metabolites such as d-glycerate-2-phosphate, pseudouridine, norcocaine nitroxide, 1-methylhistidine, 2-oxoarginine, inositol 1,3,4-triphosphate, sphinganine-1-phosphate, and 4-nitroquinoline-1-oxide demonstrated the potential of this technique to differentiate between malignant and precancerous lesions (94). Wei et al. used ultra-performance liquid chromatography combined with quadrupole/time-of-flight spectrometry (UPLC-QTOFMS) analysis to identify a signature panel of salivary metabolites (valine, lactic acid, γ-aminobutyric acid, n-eicosanoic acid, and phenylalanine) in 37 OSCC patient samples that could distinguish between OSCC from their normal counterparts with 86.5% sensitivity and 82.4% specificity. Furthermore, lactic acid and valine were significantly elevated in OSCC with respect to oral leucoplakia (OLK) with a fold change of 2.97 (p = 0.0032) and 1.60 (p = 0.0034) respectively (101). Similarly, Sugimoto et al., and Ishikawa et al. analyzed the salivary metabolomic profiles in oral cancer patients in two independent studies. These studies identified several metabolites such as cadaverine, glutamic acid, pyrrolinehydrocarboxylic acid, choline, threonine, beta-alanine, piperidine, carnitine, tryptophan, glutamine, taurine, leucine plus isoleucine, pipecolic acid, alanine, valine, and histidine that were consistently elevated in the saliva and tumor tissues of the patient samples as compared to controls (102, 103). Sugimoto’s group identified taurine and piperdine as the key oral cancer-specific markers (p < 0.05) in a pool of 69 OSCC saliva fluid samples, suggesting that metabolites in saliva can be used as biomarkers for HNC screening. Ishikawa et al. reported a high fold change value for kynurenine (FC = 38.1, p < 0.0001) (a metabolite associated with reactive oxygen species mediated stress) in tumor samples from oral cancer patients. Collectively these findings suggest that salivary metabolites reflect changes in metabolites found in tumor tissues and thus could be used for diagnosis and prognosis of oral cancers (101, 102).

Among these differentially expressed metabolites, several studies observed significantly higher levels of salivary polyamine in oral cancer patients which showed a clinical association with tumor invasion and metastasis (102). A study conducted by Hsu et al. confirmed the elevation of polyamine along with its intermediate metabolites and demonstrated a vital involvement of polyamine pathway in oral cancer progression (103, 104). These findings highlight the importance of polyamine homeostasis and its clinical utility in identifying and understanding tumor progression.

Although many studies have successfully utilized salivary metabolomics to detect HNC, inconsistency in saliva/serum derived metabolite profiles hampers the clinical utility of this approach (105). To resolve this, more evidence using larger patient cohorts is warranted. Additionally, establishing standardized protocols, analyzing intracellular metabolites and their role in HNC and understanding the underlying mechanisms behind metabolomic alterations are required in order to identify genes or proteins affected by metabolomic changes. The salivary metabolites profile tends to fluctuate as it is highly responsive to various conditions including stress. Such factors need to be accounted for as they directly impact the reproducibility of the results as well as the sample collection protocol. Salivary metabolomics is still at a nascent stage and may develop into a diagnostic tool for early detection of oral cancer.

## Salivary Microbiome

Recent studies have highlighted the role of oral microbiome in the development, progression and treatment monitoring of HNC (106). Moreover, oral microbiota has also been reported to influence salivary metabolomic profiles of HNC patients (107). Studies based on identification of bacterial spectra on the surface of OSCC mucosa in comparison to normal oral mucosa of patients revealed that there was a predominance of anaerobic pathogens in OSCC patients, compared to normal oral mucosa (108, 109). However, very little is known about the relationship between the oral microbiota and disease progression in HNC patients.

The past approaches for identification of bacterial taxa were culture dependent. However the diversity of the oral microbiome cannot be completely identified by these approaches. PCR technology and DNA-DNA hybridization methods are commonly used to describe oral microflora. However this experimental design can only identify limited changes in the microflora of a tissue (110–112). With the emergence of NGS technology, rRNA sequencing is promoted to discover the associations between microbial flora and HNC.

Pushalkar et al. examined the saliva microbiome of OSCC patients and suggested its potential application as a diagnostic tool (113). A 16S rRNA gene sequencing study on Caucasian participants found that a panel of Capnocytophaga, Corynebacterium, Porphyromonas, Haemophilus, Oribacterium, Rothia, and Paludibacter could discriminate between patients with oropharyngeal cancers and oral cavity cancers from age-matched controls (p<0.05) (114). A recent study demonstrated that an elevated presence of Capnocytophaga (AUC= 0.81 p<0.05) in saliva could be used as a probable screening tool for prognosis and diagnosis of HNC patients (115). Similarly, abundance of Dialister (p<0.05) in HNC patients correlated with aggressive laryngeal and oral tumors (116). Collectively, these studies suggest that salivary microbiota maybe useful in diagnosis and early detection of HNC.

The comprehensive role of oral microbiome in HNC development and progression is still at a nascent stage, but has been explored considerably in the last decade. However, it is still difficult to understand the exact mechanisms by which the oral microbiome contributes to HNC pathogenesis. Recently, data that links specific microbiome species to HNC aetiopathogenesis has been reported (106); however, studies based on longitudinal time frames with larger patient cohorts are needed. Longitudinal studies are critical in evaluating the dynamic nature of salivary oral microbiome before, during and after HNC development. Further research along these lines for identifying microbial biomarkers involved in tumor progression may assist in better understanding of the process of tumorigenesis and development of personalized treatments for better patient management in HNC.

## Circulating Tumor Cells (CTCs)

The tumor mass tends to shed a large number of cells through the process of apoptosis/necrosis. These cells are known as Circulating Tumor Cells (CTCs) that have the potential to create metastatic niches (117) by migrating to adjacent or distant tissues through the blood or lymphatic system. Thus, these cells are considered as seeds of metastasis or risk predictors of disease aggressiveness. CTCs have a promising role in early risk prediction, disease progression and therapeutic monitoring, and as potential drug targets (118).

CTC detection is a two-step process that involves an initial enrichment step followed by a detection step. The enrichment process comprises of two alternative approaches namely –(i) negative depletion: which focuses on removal of undesired cells (RBCs and lymphocytes) either *via* lysis or by immuno-magnetic bead-based depletion of CD45+ leukocytes; and (ii) positive selection: that involves isolation of epithelial cells using surface markers like epithelial cell adhesion molecule (EpCAM) or cytokeratins in order to distinguish the CTCs from contaminating leukocytes. The subsequent detection step is carried out using techniques ranging from quantitative PCR (qPCR) and digital PCR (dPCR) for mutational profiling to whole-genome sequencing, fluorescence *in situ* hybridization (FISH) based cytogenetic analysis and targeted NGS (119, 120). Targeted NGS-based detection of CTCs is a relatively recent advancement and is being explored for various types of cancers, including HNC (121). Immunocytochemistry (122) and flow cytometry (123) are used for single-CTC analysis but a major drawback of these two techniques is their poor multiplexing capacity. To overcome this limitation new technologies are emerging such as single-cell Western Blotting (scWB), a microfluidics-based technique used to evaluate protein levels in metastatic cancers (124). In addition, CellSearch^®^ is an EpCAM-based CTC detection system that is the only system clinically approved by the FDA for enumerating epithelial CTCs. Recent studies have highlighted the heterogeneity of CTC populations and CellSearch^®^ fails to detect CTCs with low or no expression of EpCAM and is unable to detect non epithelial tumors like sarcomas or other mesenchymal tumors. This shortcoming is overcome by using antigen-independent systems that identify CTCs based on their biophysical characteristics like density, size, and electrical properties.

CTCs can predict the risk of metastasis in HNC patients even before clinical examination (125). Hence, they may be useful for risk prediction in HNC. The presence of CTCs has been detected in saliva, however, the current landscape of CTC-based studies in HNSCC have utilized blood/plasma/serum-derived samples. Moreover, CTC evaluation is a challenge in saliva due to their limited numbers which makes isolation and detection difficult (126). The feasibility of EpCAM markers in salivary detection of CTCs, remains uncertain because of the shedding of normal epithelial cells along with cancerous cells in saliva. Nonetheless, existing studies have shown promising potential of circulatory CTCs for diagnosis, prognosis, and therapeutic monitoring in HNSCC, which suggests that further research can lead to better prospects for salivary CTCs in HNC (127–130).

## Discussion

Several studies conducted in the last decade demonstrated the plausibility of identification of potential biomarkers from biofluids and their relevance in clinical settings. Liquid biopsy has paved the way for early diagnosis and prognosis, recurrence and therapy monitoring as well as screening of high-risk populations. Although blood-based liquid biopsies have been the utmost common avenue of research, the use of salivary or oral rinse-based liquid biopsies for HNC offer a unique opportunity, as these cancers are of upper aerodigestive mucosal origin and can shed tumor cells, tumor DNA, and EVs directly into saliva. Moreover, this biopsy approach is minimally invasive, entails analysis of various circulating biomarkers and enables real time monitoring of tumor progression using repetitive testing. Such real time monitoring is simply not possible with traditional biopsies. As cancer treatment moves toward an attention on targeted precision medicine, liquid biopsy has the potential to guide such treatments based on real time monitoring of patients. The current review highlights new technological advancements and potential clinical applications of saliva as a liquid biopsy tool in HNC. CTCs, ctNAs, EVs, and salivary metabolome can yield useful biomarkers using non-invasive techniques. These biomarkers could reflect actual tumor biomarkers. The copious work, involving an extensive variety of assays based on diverse principles, has been quite productive in terms of utility of these biomarkers in diagnosis and disease monitoring of head and neck cancers. However, a major obstacle for all biomolecules in liquid biopsy remains the relatively low and fluctuating concentration derived from a tumor against the background of normal counterparts; in most patient samples. Such hurdles are tackled using the approaches highlighted in the technologies addressed above. These methods are presently sensitive enough to detect and analyze very rare mutation events. Nevertheless, it is crucial that laboratories working with such techniques must be consistent in their methodologies to avoid inaccurate results. Though passé, the association of a needle in a haystack relates and is fitting for each of these practices.

The investigation of ctNAs and EVs has benefitted from advances in the field of enrichment former to the analytical procedures. While at a nascent stage, reports have revealed that isolation and enrichment techniques will be an important asset in refining nucleic acid-based assays and as an individual diagnostic in the future.

Evidently, EVs have various advantages for prognosis and diagnosis. They aid in extraction of high-quality RNA from fresh or frozen saliva, thus enhancing the scope of detectable mutations that comprise of splice variants, mutations, fusions along with expression-based assays for mRNA, microRNA, lncRNA and other non-coding RNAs. ctDNA contains all genes at an equal level, while RNA originating from a highly expressed gene could be present in thousands of copies/cells. Nevertheless, as mutations exist on both ctRNA (dying/apoptotic process) and exosome RNA (living process), developing a platform that can aid in both will have palpable advantages for detecting rare mutations. This can be of great help in the case of patients who do not have an ample quantity of mutated nucleic acid in circulatory fluid. Moreover, as DNA mutations will only notify limited information of the disease, investigating RNA expression in biofluids such as saliva can further help in understanding the processes within the HNC patient. Although saliva is a promising source of all these biomolecules it is currently unclear which one of these (ctNAs, EVs or metabolites) will eventually be useful in early diagnosis, tumor prognosis and real time therapeutic monitoring. It is entirely possible that each of these end points require monitoring different biomolecule levels. Advances in technologies for sensitive, robust and inexpensive detection of such biomolecules will enable the use of saliva based liquid biopsies in routine clinical use.

Cancer is a multifaceted and dynamic disease that can undergo quick changes. To copiously deliver on the assurance and surety of personalized medicine, development of reliable non-invasive avenues for the diagnosis, prognosis, patient stratification and treatment response monitoring are paramount. Further studies in clinical settings and in large patient cohorts with well-annotated data are needed to validate the salivary transcriptomic, genomic and proteomic data. The several liquid biopsy platforms explained in this review have the ability to add immense value to the care of cancer patients.

## Authors Contributions

**AP** and **SP** participated in the literature analysis. **AP** and **PP** searched the literature, drafted the manuscript and created the figures and tables. **SP** and **VT** designed, conceptualized, finalized and contributed to the critical review of the manuscript. All authors contributed to the article and approved the submission.

## Conflict of Interest

The authors declare that the research was conducted in the absence of any commercial or financial relationships that could be construed as a potential conflict of interest.

## Publisher’s Note

All claims expressed in this article are solely those of the authors and do not necessarily represent those of their affiliated organizations, or those of the publisher, the editors and the reviewers. Any product that may be evaluated in this article, or claim that may be made by its manufacturer, is not guaranteed or endorsed by the publisher.
